# Variation in claw morphology among the digits of Bent-toed geckos (*Cyrtodactylus*: Gekkota: Gekkonidae)

**DOI:** 10.1186/s40850-023-00180-x

**Published:** 2023-09-08

**Authors:** Jendrian Riedel, Melinda Klemm, Timothy Higham, L. Lee Grismer, Thomas Ziegler, Anthony Russell, Dennis Rödder, Klaus Reinhold

**Affiliations:** 1 Section Herpetology, Leibniz Institute for the Analysis of Biodiversity Change – Museum Koenig, Bonn, Germany; 2 Department of Evolutionary Biology, Bielefeld University, Bielefeld, Germany; 3 Department of Evolution, Ecology, and Organismal Biology, University of California, Riverside, CA USA; 4 Department of Biology, La Sierra University, Riverside, CA USA; 5 Department of Herpetology, San Diego Natural History Museum, San Diego, CA USA; 6 Cologne Zoo, Cologne, Germany; 7 Institute of Zoology, University of Cologne, Cologne, Germany; 8 Department of Biological Sciences, University of Calgary, Calgary, AB Canada

**Keywords:** Claw, *Cyrtodactylus*, Morphology

## Abstract

**Background:**

Ecomorphological studies of lizards have increasingly employed comparison of claw morphology among species in relation to spatial niche use. Typically, such studies focus on digit IV of the autopodia, especially the pes. Uniformity of claw morphology among digits is more often implicitly assumed than tested.

**Results:**

Using four species of *Cyrtodactylus*, comprising two generalist and two scansorial taxa that use different substrates, we examined whether claw morphology is uniform among digits and among species. We found that, within each species, ventral claw curvature is uniform across all digits whereas there are small but insignificant differences in ventral claw length and claw depth. The claws of the pes of each species are longer and deeper than those of the corresponding digits of the manus. The claw of digit I of each species is significantly shorter and shallower on both autopodia compared to those on digits IV and V (digit I, including its claw, is idiosyncratically variable among lizards in general).

**Conclusions:**

We conclude that digit IV is an adequate representative of claw form in each species and exhibits variation among species, thereby serving as an exemplar for use in studies of potential discrimination between ecomorphological types in studies of *Cyrtodactylus*.

**Supplementary Information:**

The online version contains supplementary material available at 10.1186/s40850-023-00180-x.

## Background

Ecological adaptations of the locomotor apparatus to different spatial microhabitats are widespread across animals [1–3], including morphological variation exhibited by claws [4, 5]. Claws are critical for attachment to surfaces, including the penetration of soft surfaces, mechanical interlocking with rough surfaces, or simply by enhancing friction [6]. In some cases, such variation can be associated with adaptive radiations, such as in the well-studied ecomorphs of *Anolis* lizards [7–9]. Terrestrial species, particularly cursorial ones occupying open habitats, often have relatively longer and less tightly curved claws, while scansorial species often have more tightly curved claws that are deeper at their base [10, 11]. The longer and more attenuated claws putatively increase the lever arm of the limbs and thus contribute to faster running speed. In contrast, the combination of tighter curvature and a deeper claw base should promote more effective interlocking with, or piercing of, the substrate by increasing claw stability [4, 10, 12] and providing for a shorter out-lever arm over which claw flexor tendons impart their forces.

Such associations have been studied in many taxa but most frequently in squamates and birds (which, except for hoatzins, possess claws only on the hind limbs) [12, 13]. Such studies generally focus on a single digit, usually digit III in birds and digit IV in lizards, arguing that these are generally the longest and most prominent digits in the respective clade (thus potentially incurring smaller measurement errors) and are those most reflective of the mode of locomotion (e.g., [5, 14, 15]). Furthermore, although some studies of lizards incorporate the claws of both the fore and hind limbs, many focus solely on the pes due to its greater contribution to thrust production in cursorial locomotion [16].

While such selective approaches are understandable, the underlying, but unstated, assumptions that the relative claw proportions are uniform (or at least highly similar) among digits within an autopodium and between the manus and pes have seldom been tested. Studies that have examined among-digit variation of claw form have engendered different conclusions, depending on the clades being examined. Studies of lacertid and varanid lizards that include data from digit IV of the manus and pes revealed no differences between them [15, 17]. In contrast, examination of neotropical iguanian lizards [10] yielded differential ecomorphological associations among digits with regard to claw length and depth, revealing that high trait values were associated with different microhabitats for different digits. Similarly, Birn-Jeffery et al. [12] found differences among digits for inner and outer claw curvature and for mid-point claw depth in their study of birds and lizards. These examples indicate that uniformity of claw morphology among digits and autopodia (manus and pes) should not be assumed because differences may occur and may even be of adaptive value in some cases, such as the extinct deinonychosaurian dinosaurs and modern seriema birds [18].

With over 340 species, *Cyrtodactylus* Gray 1827 is the most speciose genus within gekkotans [19]. All species are assignable to one of ten different ecotypes [20, 21]. And recent research suggests that at least some of these ecotypes exhibit distinct morphological associations in terms of limb and body proportions. This indicates that they might constitute distinct ecomorphs [22] adapted to their respective microhabitats through different aspects of their locomotor morphology [23, 24].

In light of this, and as a prelude to a more extensive investigation of the morphometrics of ecomorphological variability in the gekkonid genus *Cyrtodactylus*, we examine the potential for uniformity of claw form among digits within and between the autopodia of four of its species. Our overall goal is to assess whether digit IV of the manus and pes can be employed as reliable indicators of claw morphology within and among species.

## Results

**Fig. 1:**
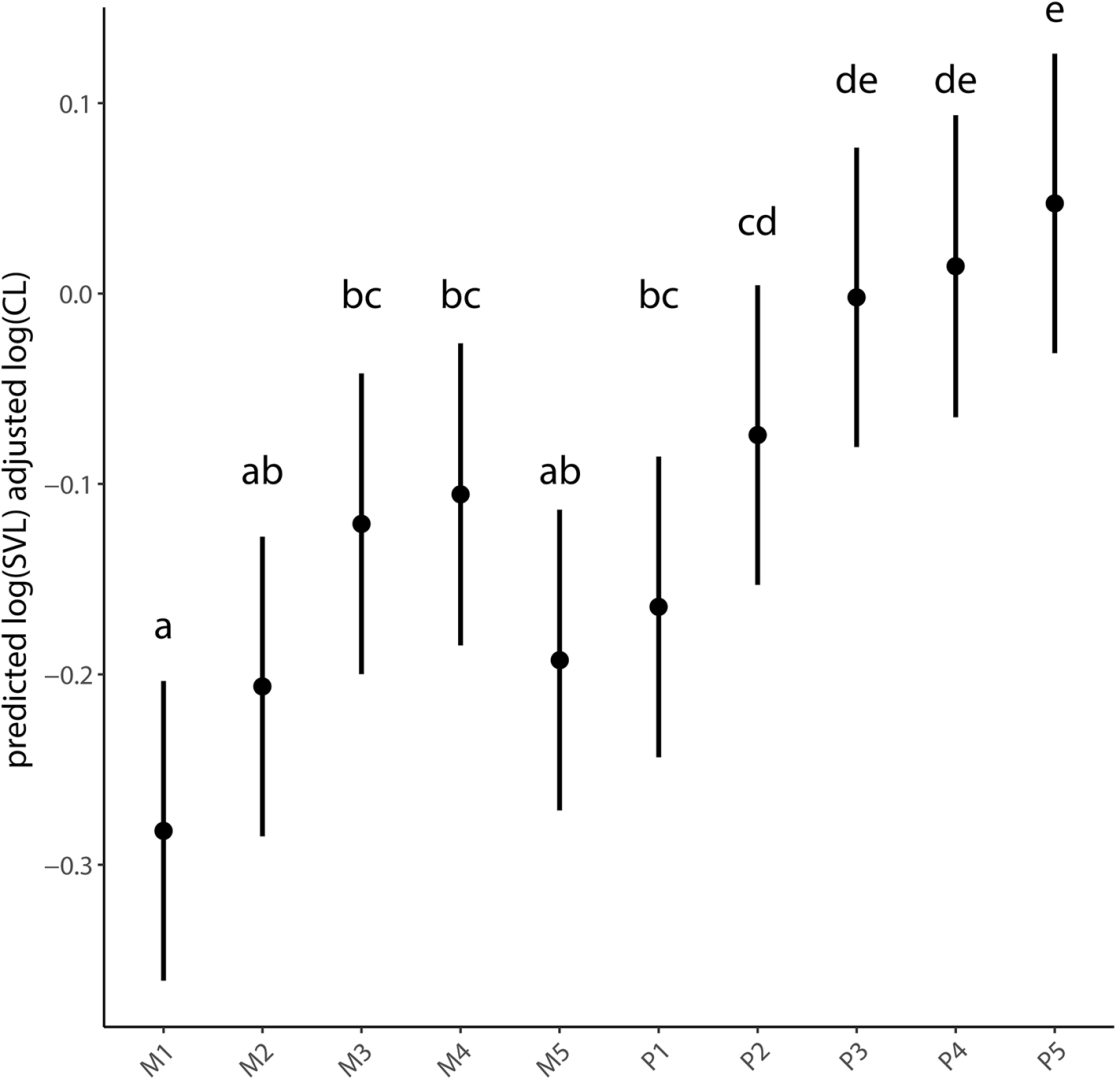
Predicted mean vales and 95% confidence intervals for size-adjusted claw length (on a log scale) for all species. Significant differences (a-e) are shown among digits. CL = claw length, SVL = snout-vent-length. M1-M5: pooled (left and right) manual digits I to V, P1-P5: pooled (left and right) pedal digits I to V

Claw length across all digits and all species was positively correlated with SVL (snout-to-vent length) (F_1,1_ = 13.210, *p* = 0.003). Both species (F_1,3_ = 8.488, *p* = 0.002) and digit (F_1,9_ = 24.236, *p* < 0.001) showed significant differences in size-adjusted claw length. Across species, post hoc comparisons among pedal digits revealed a gradual increase of claw length from digit I to V, with the claw of digit I being significantly shorter than that of digits II-V, and that of digit II being significantly shorter than that of digit V (Fig. 1). Within the manus there was a gradual increase in claw length from digit I to IV, followed by a minor decrease to digit V (Fig. 1). The claw of manual digit I was significantly shorter than that of digits III and IV.

Across species, the claws of digits III-V of the pes were significantly longer than those of all digits of the manus, whereas the claw of digit II of the pes was significantly longer than that of all digits of the manus except for III and IV. The claw of the first digit of the pes was significantly longer than that of the first digit of the manus (Fig. 1) but was not longer than that of any other digits of the manus or pes.

A post hoc comparison among species revealed that *C. annulatus* had overall significantly longer claws than *C. zebraicus,* with the other two species falling in between them (Supplement file 1 – SF2).

**Fig. 2:**
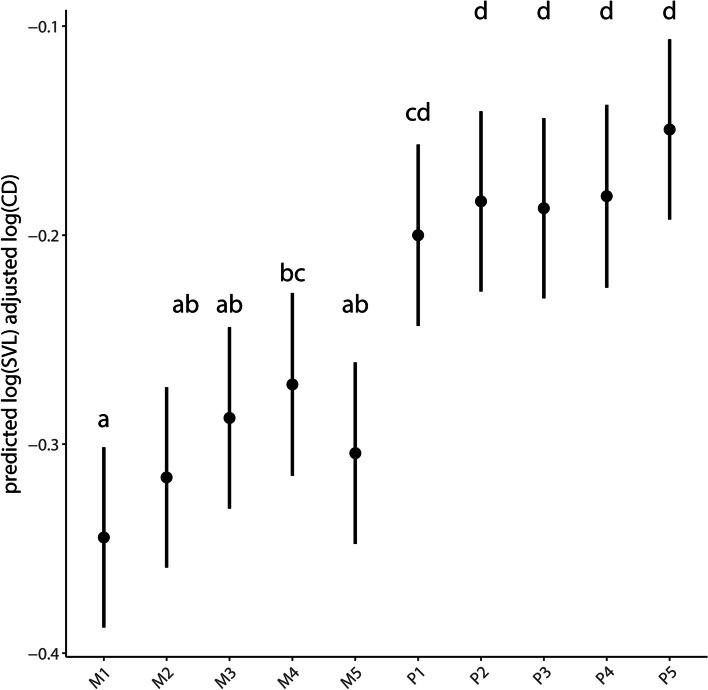
Predicted mean vales and 95% confidence intervals for size adjusted claw depth (on a log scale) for all species. Significant differences (**a**-**d**) are shown among digits. CD = claw depth, remaining abbreviations as in Fig. 1

Claw depth across digits and species was positively correlated with SVL (F_1,1_ = 45.469, *p* < 0.001), and both fixed effects showed significant differences (digit: F_1,9_ = 19.190, *p* < 0.001, species: F_1,3_ = 5.929, *p* < 0.01) in size-adjusted claw depth. Post hoc comparison among digits (across all species) showed no significant differences within the digits of the pes and that the claws of all digits of the pes, with one exception, were significantly deeper than those of the corresponding digits of the manus. The depth of the claw of digit I of the pes overlapped with that of digit IV of the manus (Fig. 2). Within the manus, the claw of digit IV was significantly deeper than that of digit I, with the claw depths of all other digits overlapping with both of these. The results of the post hoc comparisons among species were similar to those for claw length (Supplement file 1 – SF3).

Claw curvature differed significantly among species (F_1,3_ = 14.733, *p* < 0.001), but did not differ among digits for all species (F_1,9_ = 0.857, *p* = 0.554). The claws on all digits of the two scansorial species, *C. tiomanensis* and *C. consobrinus,* were more tightly curved than those of the generalists *C. annulatus* and *C. zebraicus* (Supplement file 1 – SF4).

## Discussion

Overall, we found that claw curvature differed among the four species of *Cyrtodactylus*, but was invariant for all five digits of all autopodia for all species. Both claw length and depth showed varying degrees of differentiation among digits and among species. The claws on the pedal digits tended to be longer and deeper than those of the corresponding digits of the manus in all species, although the differences were more pronounced for claw depth than length. Within each autopodium of all species, claw length and depth gradually increased from digit I to digit IV of the manus and from digit I to digit V of the pes, although the differences were not significant except for the digits occupying the endpoints of the digital arcades (Figs. 1 and 2).

These subtle differences correspond to the overall morphology of the digits in squamate reptiles. The phalangeal formula for *Cyrtodactylus* is 2–3-4–5-4 for the pes and 2–3-4–5-3 for the manus, which is the plesiomophic phalangeal formula for lizards [25, 26]. Furthermore, digit I of both the manus and pes is also the digit that most frequently exhibits claw reduction or complete loss in geckos that have fully developed adhesive pads on that digit, with digits II and V of the manus and pes exhibiting claw reduction and loss less frequently [27, 28]. Although some lineages also exhibit claw reduction or loss on all digits, the lower frequency of claw reduction in the central digits, III and IV further indicates that the minor trends observed here are in accord with general developmental and evolutionary trends in geckos.

From a biomechanical perspective, the hindlimbs of lizards are the major contributors to locomotor thrust and are, therefore, generally more robust when compared to the forelimbs. Digit IV of the pes is the last digit to lose contact with the substratum during level running in lizards and thus serves as the major lever arm for acceleration [16, 26]. For climbing species, tightness of claw curvature is putatively the most important claw trait [15] and was found to be uniform among digits in all species in our sample, supporting a more ubiquitous involvement of the claws in climbing for geckos [29]. Thus, the minor differences observed in claw length and claw depth among digits might be simply associated with overall digit length and phalangeal number, but could also be attributable to other developmental constraints as well as partially reflecting their differential contribution to at least some modes of locomotion [30].

The four species examined in this study showed differentiation in claw parameters among species. When considered with their spatial microhabitat use, our results suggest ecomorphological differences in claw morphology might occur in this genus. Our limited sample size in terms of species, however, renders conclusions based on habitat premature and we therefore refrain from discussing further potential differences among species here. More extensive exploration and examination of claw morphology in *Cyrtodactylus* is needed to thoroughly test possible ecological associations of claw morphology and spatial niche use.

## Conclusion

We can conclude that claw curvature for our sample of four species of *Cyrtodactylus* geckos is indeed uniform among digits within each species, whereas claw length and height are highly similar with only subtle differences among digits within each species. These subtle differences are in line with proportional differences in digit morphology among the digits of lizards in general [26], and also in line with slightly differential contributions to some locomotor modes, such as digit IV (at least for the pes) contributing somewhat disproportionately more to cursorial locomotion [16] and being somewhat longer and more robust than the less pronounced more medial (inner) digits. The latter functional association implies that if ecomorphological differences among species exist, these differences might be more pronounced in digit IV compared to the inner digits for those claw dimensions showing minor differences among digits. Thus, digit IV of both the manus and pes is suitable for representing overall claw morphology in the genus *Cyrtodactylus* for ecomorphological studies.

## Methods

Our study included four focal species, each from a unique species group [20]. Because almost 50% of *Cyrtodacylus* species have been assigned to a generalist ecotype [20], characterized by using both the ground and a variety of rocks and the lower reaches of trees as habitat, we selected two generalist species for investigation: the Philippine species *Cyrtodactylus annulatus* (Taylor, 1915) from the *philippinicus* group and the Thai species *C. zebraicus* Taylor, 1962 from the *oldhami* group [20, 31]. For habitat specialists, we chose two scansorial species: the trunk specialist *C. consobrinus* (Peters, 1871) from the *malayanus* group and the granite specialist *C. tiomanensis* Das and Lim, 2000 [32] from the *agamensis* group, both of which hail from peninsular Malaysia [20, 31].

**Fig. 3:**
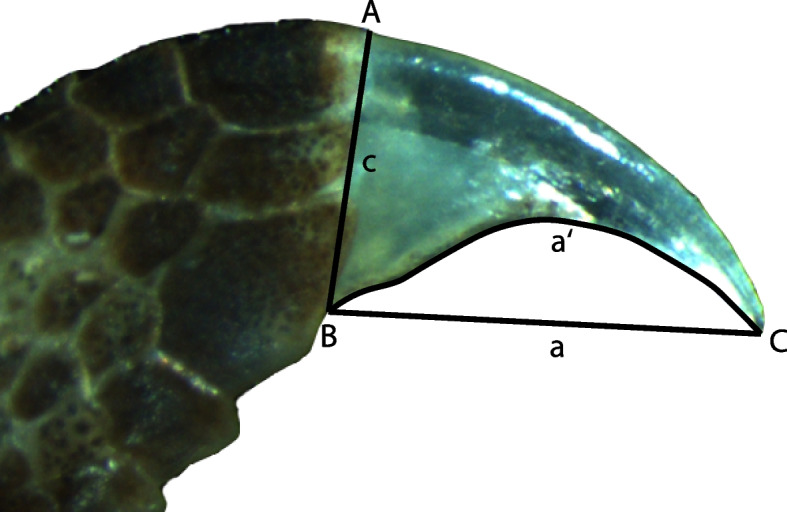
Illustration of the claw parameters measured. Claw length was measured as the length of line a from the claw tip (**C**) to the ventral claw base (**B**) and claw depth as the length of line c from the dorsal (**A**) to the ventral (**B**) claw base. Claw curvature was calculated as the claw length a divided by the length of the inner claw arc a´

We examined five ethanol-preserved adult specimens of each species from the collection of the Museum Koenig in Bonn (Germany) (Table 1). Each claw of all 10 digits (manus and pes) was photographed (unless otherwise noted in Table 1) in lateral view, accompanied by a scale bar, using a microscope (Motic SMZ-171, Motic, Barcelona) at a magnification of 5x. Thirteen claws from eight specimens had to be excluded due to claw damage (Table 1). Distance were then measured in ImageJ v1.53 k using the straight line tool for linear measurements and the segmented line tool for curved lines [33]. For all four species we recorded claw depth (dorsoventrally at the claw base, Fig. 3 segment c), ventral claw length (Fig. 3, segment a) and inner claw curvature. The former two measurements were taken directly as linear measurements whereas claw curvature was calculated as the ratio of linear claw length (Fig. 3, segment a) divided by the length of a curved line following the ventral claw arc (Fig. 3, segment a’). Additionally, we measured snout-vent-length (SVL) for all specimens directly from the specimens using a digital calliper (Model 108–4500, Imatec, Luxembourg) to account for specimen size in our analysis. To compensate for uncertainty in measurement accuracy, each measurement was taken three times and mean values per digit and specimen were used in the analysis.

**Table 1:** Specimens sampled for this study. The last column lists claws that were damaged and thus excluded from measurements (L = left, R = right, M = manus, P = pes, I-V: digit number; m = male, f = female)

Species	Specimen Number	Sex	Damaged claws
*C. zebraicus*	ZFMK 43835	f	RP I, RP III, RM IV
*C. zebraicus*	ZFMK 43836	f	LP V
*C. zebraicus*	ZFMK 43837	m	LP I, RP V
*C. zebraicus*	ZFMK 43838	m	RP I
*C. zebraicus*	ZFMK 43876	m	
*C. annulatus*	ZFMK 52339	m	LP I
*C. annulatus*	ZFMK 52340	m	
*C. annulatus*	ZFMK 52341	f	LM IV
*C. annulatus*	ZFMK 52342	f	
*C. annulatus*	ZFMK 52343	f	
*C. tiomanensis*	ZFMK 84878	m	
*C. tiomanensis*	ZFMK 84879	m	
*C. tiomanensis*	ZFMK 84880	f	
*C. tiomanensis*	ZFMK 84881	f	
*C. tiomanensis*	ZFMK 84882	f	
*C. consobrinus*	ZFMK 86720	m	
*C. consobrinus*	ZFMK 86721	m	LM III–IV
*C. consobrinus*	ZFMK 86722	m	
*C. consobrinus*	ZFMK 86723	f	
*C. consobrinus*	ZFMK 86724	f	LP IV, RP IV

All statistical analyses were conducted in RStudio v4.21 [34]. All datapoints were log-transformed to improve normality and reduce heteroscedasticity. Linear mixed effect models were constructed using the ‘lmer’ function of the package lme4 [35]. First, we tested each of the three claw parameters individually for bilateral symmetry. As we did not detect significant differences between body sides (Supplement file 1—SM1) we were able to pool our measurements for corresponding digits on the left and right side of the body for the main analysis. Thus, further comparisons are made between manual digits I-V and pedal digits I-V, with no indication of side. To test whether the three claw parameters were different within species among digits, and among species for corresponding digits, each parameter was analysed separately. Digit and species were treated as fixed effects in all three models, while specimen number was added as a random effect. For claw length and claw depth, SVL was added as a fixed effect to account for differences in body size. We then applied a type II ANOVA to each model using the’ANOVA’ function of the car package [36] with an alpha level of 0.05 and tested for differences among categories in a post hoc comparison using the ‘emmeans’ function of the package Emmeans [37] for those categories and parameters with significant ANOVA results.

### Supplementary Information

**Additional file 1: Supplement SM1.** Test for bilateral symmetry. **Supplementary Figure SF1.** Box plots of the raw values for (A) claw depth, (B) claw length, and (C) claw curvature. The plot on the left shows mean values per species for each trait, while the right hand side plot shows mean per digit. The left side of the body is shown in red while the right side is shown in green. Note that these values are not size corrected. **Supplementary Figure 2.** Predicted mean vales and 95% confidence intervals for size adjusted claw length for all digits. Significant differences (a-b) are shown among species. CL = claw length, SVL = snout-vent-length. **Supplementary Figure 3.** Predicted mean vales and 95% confidence intervals for size adjusted claw depth for all digits. Significant differences (a-b) are shown among species. CD = claw depth, SVL = snout-vent-length. **Supplementary Figure 4**. Predicted mean vales and 95% confidence intervals for claw curvature (CC) for all digits. Significant differences (a-b) are shown among species.

**Additional file 2. **

## Data Availability

The raw dataset for this study is available in the supplement file 2.
