# Supplementary material for: Variation in claw morphology among the digits of Bent-toed geckos (Cyrtodactylus: Gekkota: Gekkonidae)
Source: BMC Zool. 2023 Sep 8;8:19. doi: 10.1186/s40850-023-00180-x (PMC10492352; doi:10.1186/s40850-023-00180-x)
Supplement: Supplementary file 1 — Additional file 1: Supplement SM1. Test for bilateral symmetry. Supplementary Figure SF1. Box plots of the raw values for (A) claw depth, (B) claw length, and (C) claw curvature. The plot on the left shows mean values per species for each trait, while the right hand side plot shows mean per digit. The left side of the body is shown in red while the right side is shown in green. Note that these values are not size corrected. Supplementary Figure 2. Predicted mean vales and 95% confidence intervals for size adjusted claw length for all digits. Significant differences (a-b) are shown among species. CL = claw length, SVL = snout-vent-length. Supplementary Figure 3. Predicted mean vales and 95% confidence intervals for size adjusted claw depth for all digits. Significant differences (a-b) are shown among species. CD = claw depth, SVL = snout-vent-length. Supplementary Figure 4. Predicted mean vales and 95% confidence intervals for claw curvature (CC) for all digits. Significant differences (a-b) are shown among species. [file 40850_2023_180_MOESM1_ESM.pdf]

**Supplementary Material to Variation in claw morphology among the digits of bent-toed geckos (*Cyrtodactylus*: Gekkota: Gekkonidae)**

Jendrian Riedel, Melinda Klemm, Timothy Higham, L. Lee Grismer, Thomas Ziegler, Anthony Russell, Dennis Rödder, Klaus Reinhold

## Supplement SM1 - Test for bilateral symmetry

We constructed one model for each of the three claw parameters (length, depth, and curvature) to test if they showed bilateral symmetry using the 'lmer' function of the package lme4 (Bates, Maechler, Bolber, & Walker, 2015). Each model included body side (left vs. right) as a fixed effect. The models for claw length and claw depth included body length (SVL) as an additional fixed effect. Digit (Manus I – V and pes I-V)), species and specimen number were all treated as random effects to account for potential differences among these factors. Each model was then analysed with a type II ANOVA using the 'ANOVA' function of the car package (Fox & Weisberg, 2019).

Both, claw length and claw depth were positively correlated with SVL (claw length: $F_{1,1} = 68.476$, $p < 0.001$; claw depth: $F_{1,1} = 20.180$, $p < 0.001$). Body side did not show significant differences for any of the three claw traits (claw length: $F_{1,1} = 0.806$, $p = 0.370$; claw depth: $F_{1,1} = 0.0758$, $p = 0.783$; claw curvature: $F_{1,1} = 0.140$, $p = 0.708$, see figure SF1)

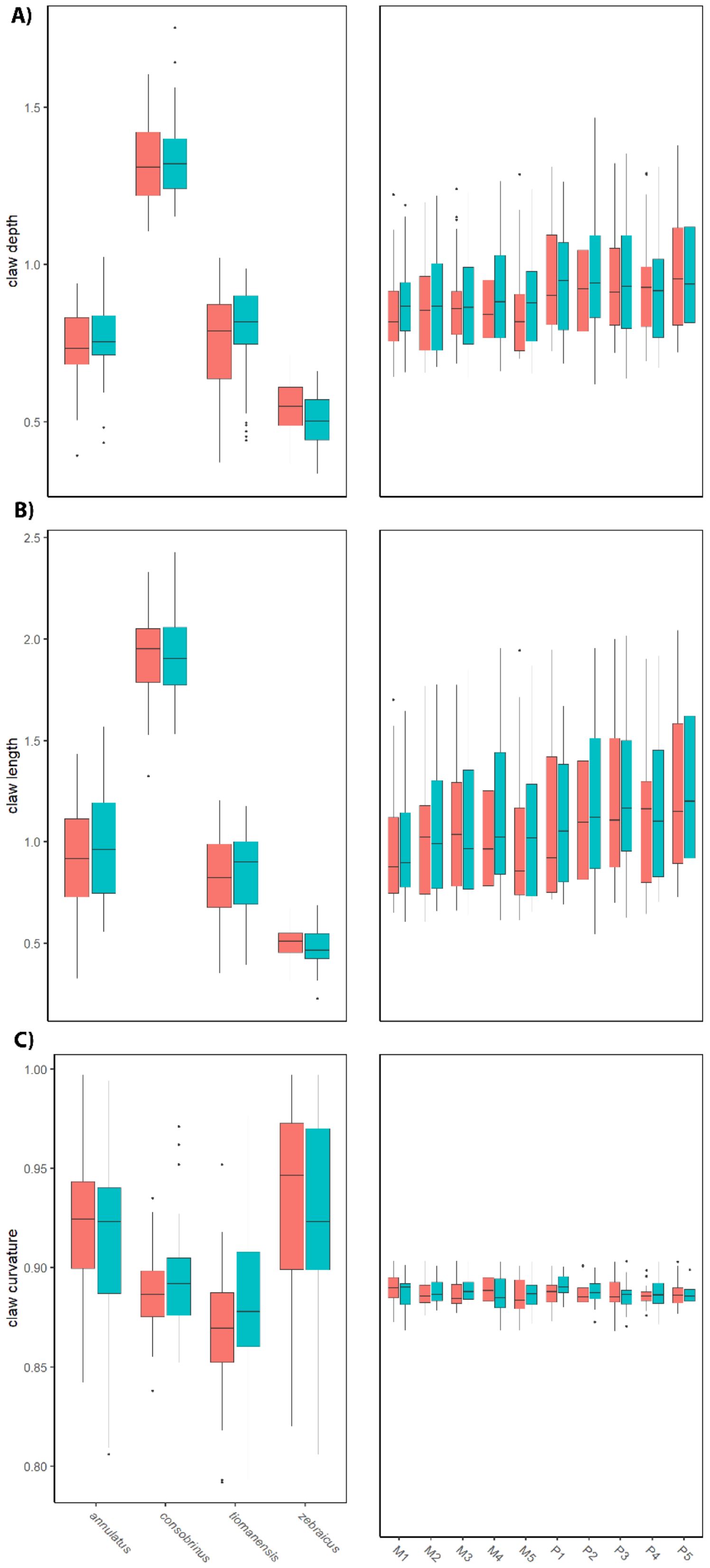


Supplementary Figure SF1: Box plots of the raw values for (A) claw depth, (B) claw length, and (C) claw curvature. The plot on the left shows mean values per species for each trait, while the right hand side plot shows mean per digit. The left side of the body is shown in red while the right side is shown in green. Note that these values are not size corrected.

Supplement SF2:

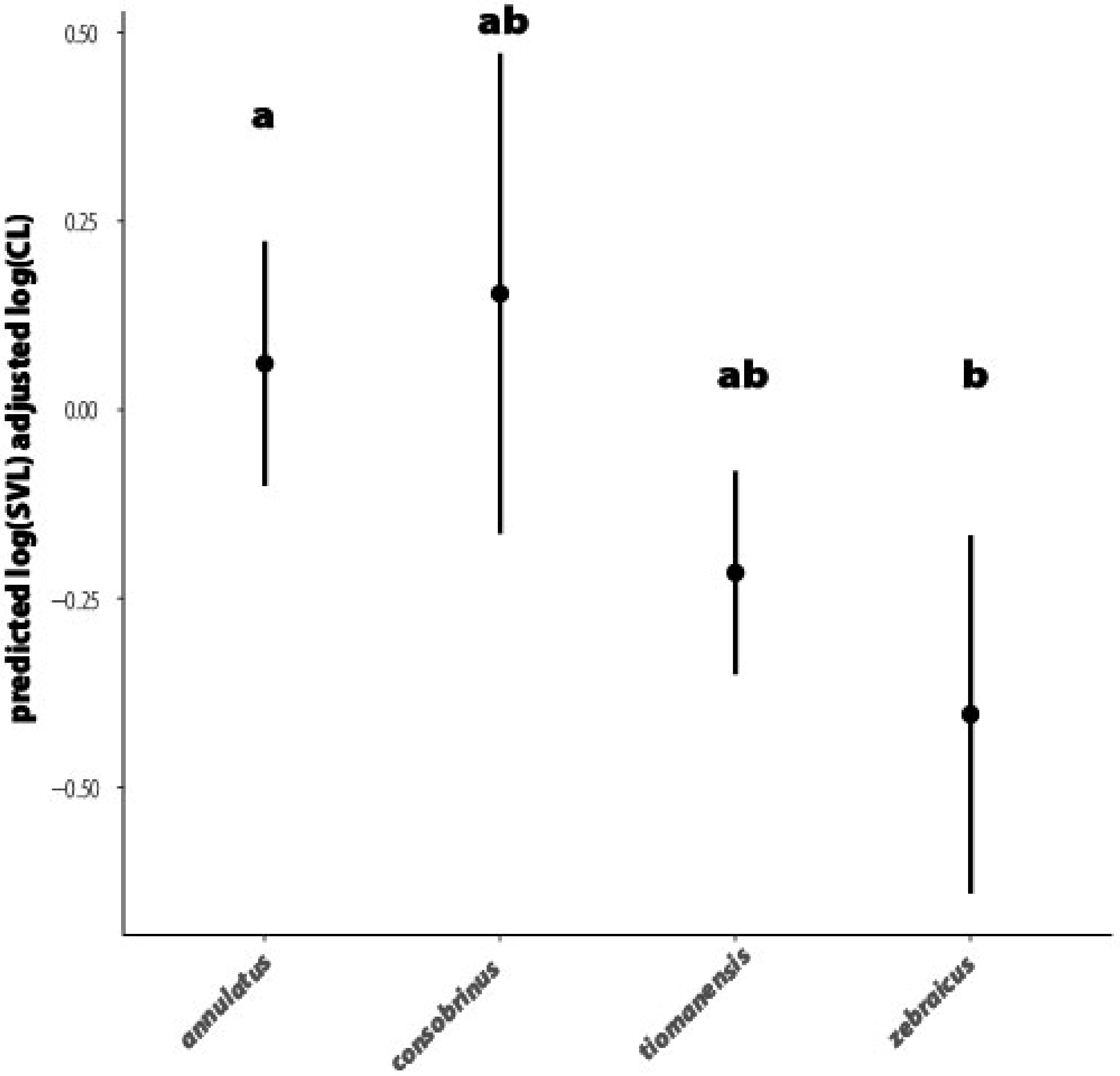


Supplementary Figure 2: Predicted mean vales and 95% confidence intervals for size adjusted claw length for all digits. Significant differences (a-b) are shown among species. CL = claw length, SVL = snout-vent-length.

Supplement SF3:

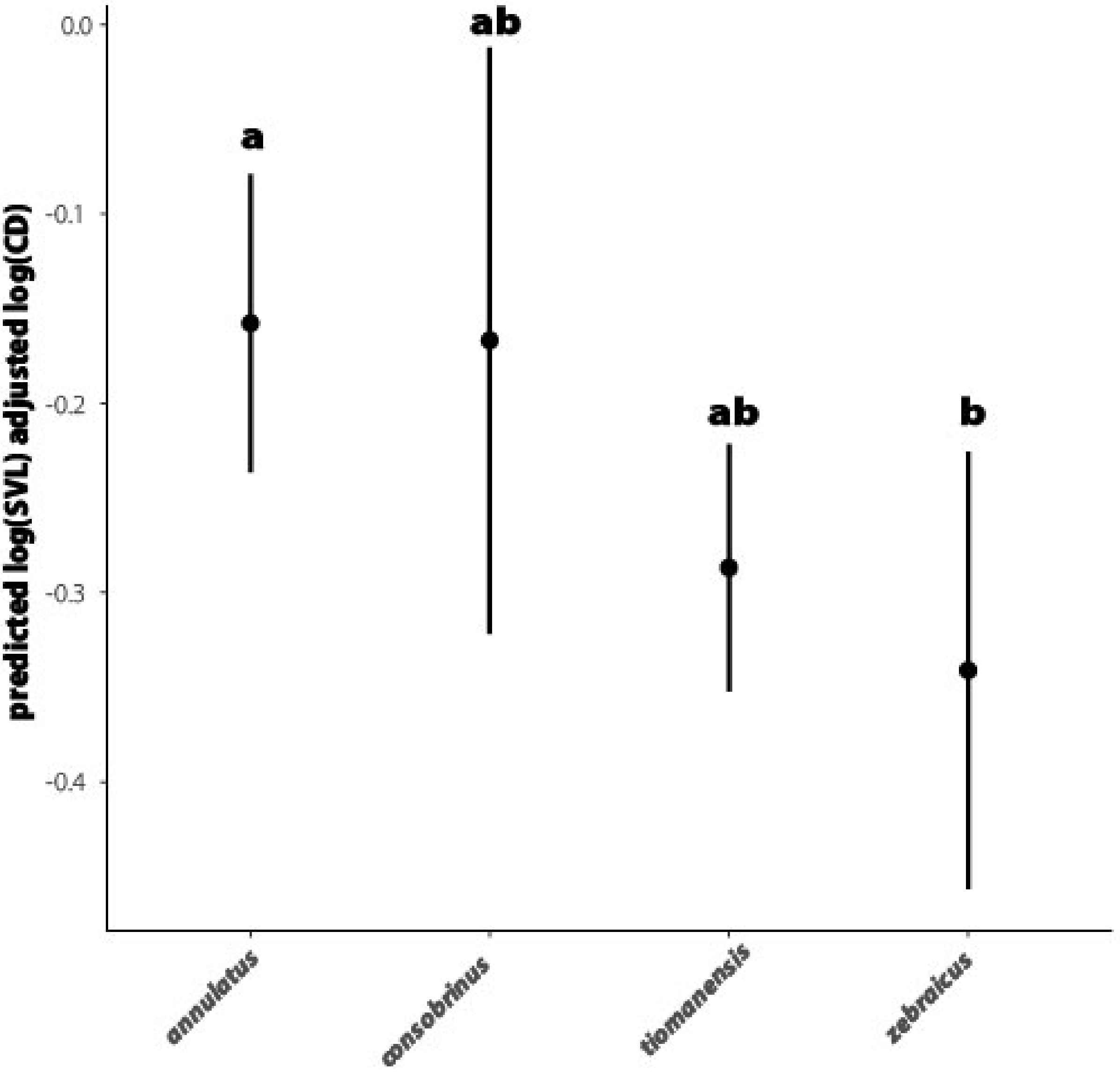


Supplementary Figure 3: Predicted mean vales and 95% confidence intervals for size adjusted claw depth for all digits. Significant differences (a-b) are shown among species. CD = claw depth, SVL = snout-vent-length.

Supplement SF4:

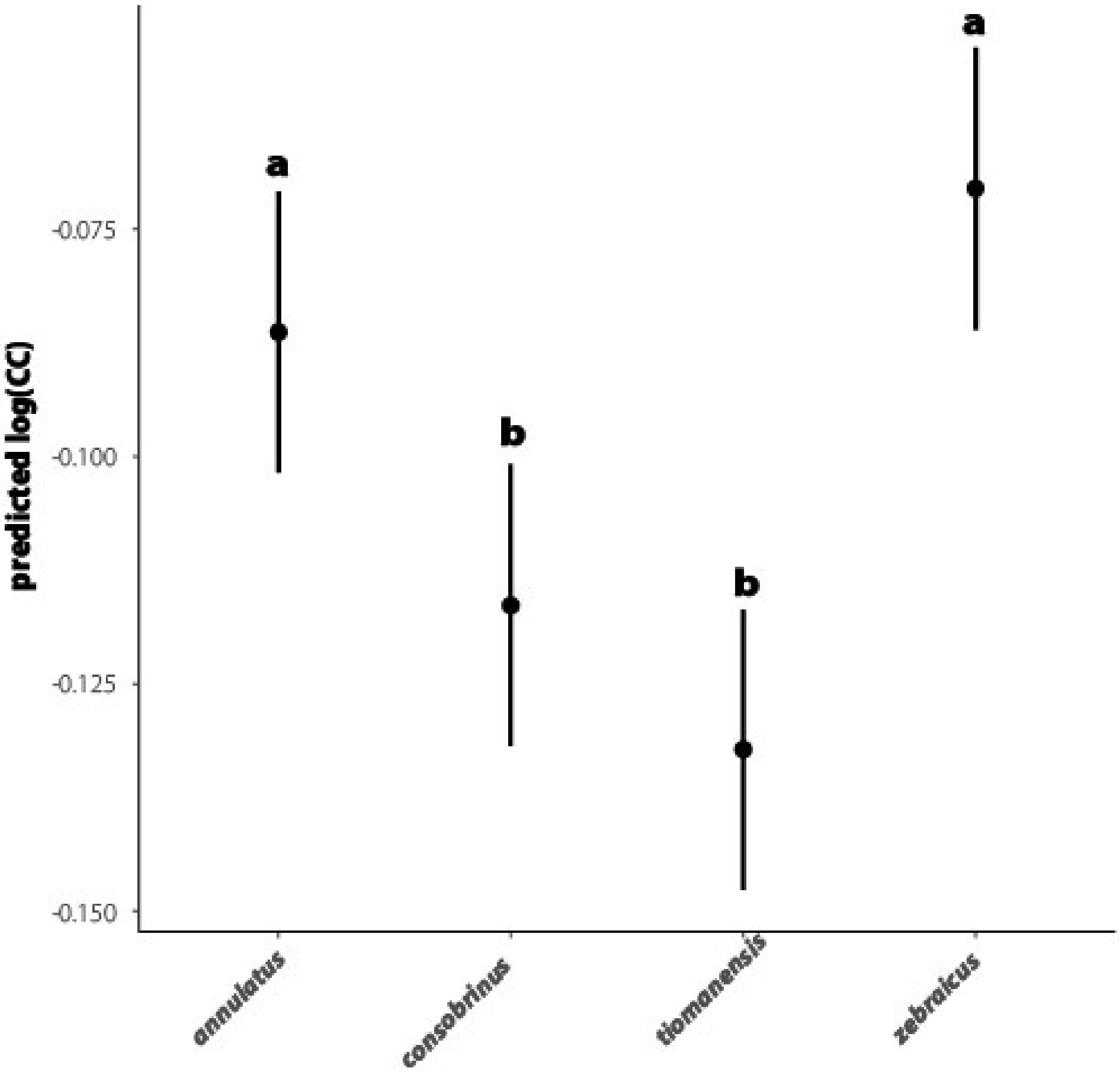


Supplementary Figure 4: Predicted mean vales and 95% confidence intervals for claw curvature (CC) for all digits. Significant differences (a-b) are shown among species.
